# Necroptosis is active and contributes to intestinal injury in a piglet model with lipopolysaccharide challenge

**DOI:** 10.1038/s41419-020-03365-1

**Published:** 2021-01-11

**Authors:** Yulan Liu, Qiao Xu, Yang Wang, Tianzeng Liang, Xiangen Li, Dan Wang, Xiuying Wang, Huiling Zhu, Kan Xiao

**Affiliations:** Hubei Key Laboratory of Animal Nutrition and Feed Science, Wuhan Polytechnic University, 430023 Wuhan, China

**Keywords:** Cell death, Transcription

## Abstract

Necroptosis, a newly discovered form of programmed cell death that combines the features of apoptosis and necrosis, is important in various physiological and pathological disorders. However, the role of necroptosis on intestinal injury during sepsis has been rarely evaluated. This study aimed to investigate the presence of necroptosis in intestinal injury, and its contribution to intestinal injury in a piglet model challenged with *Escherichia coli* lipopolysaccharide (LPS). Firstly, a typical cell necrotic phenomenon was observed in jejunum of LPS-challenged pigs by transmission electron microscope. Protein expression of necroptosis signals including receptor-interacting protein kinase (RIP) 1, RIP3, and phosphorylated mixed-lineage kinase domain-like protein (MLKL), mitochondrial proteins including phosphoglycerate mutase family member 5 (PGAM5) and dynamin-related protein 1 (DRP1), and cytoplasmic high-mobility group box 1 (HMGB1) were time-independently increased in jejunum of LPS-challenged piglets, which was accompanied by the impairment of jejunal morphology, and digestive and barrier function indicated by lower activities of jejunal disaccharidases and protein expression of jejunal tight junction proteins claudin-1 and occludin. Pro-inflammatory cytokines including tumor necrosis factor-α (TNF-α), interleukin (IL)-1β, and IL-6 were also dynamically induced in serum and jejunum of piglets after LPS challenge. Moreover, pretreatment with necrostatin-1 (Nec-1), an specific inhibitor of necroptosis, inhibited necroptosis indicated by decreased necrotic ultrastructural changes and decreased protein expression of RIP1, RIP3, and phosphorylated MLKL as well as PGAM5, DRP1, and cytoplasmic HMGB1. Nec-1 pretreatment reduced jejunal morphological injury, and improved digestive and barrier function. Nec-1 pretreatment also decreased the levels of serum and jejunal pro-inflammatory cytokines and the numbers of jejunal macrophages and monocytes. These findings indicate for the first time that necroptosis is present and contributes to LPS-induced intestinal injury. Nec-1 may have a preventive effect on intestinal injury during sepsis.

## Introduction

Sepsis is a critical and life-threatening clinical condition, and is characteristic of a comprehensive systemic inflammatory response to infection^1^. As sepsis progresses, severe pathophysiological conditions including multiple organ failure, shock, and death, commonly occur^1^. Sepsis is closely associated with damage to the intestinal barrier^2,3^. Damage of the intestinal barrier increases gut permeability, leads to bacterial translocation and exacerbates the injury of intestinal integrity, and subsequently leads to systemic inflammatory response syndrome, multiple organ failure, and septic shock^4,5^. So, understanding the mechanisms of disturbances in intestinal integrity is critical for the prevention and treatment of sepsis.

The structural integrity of the intestine and an effective intestinal barrier mainly depend on the balance between epithelial cell proliferation and death^6^. So, intestinal epithelial cell death has to be tightly controlled and irregularities may lead to pathologies^6^. Compelling evidence has shown that excessive cell death in the intestinal epithelium can induce intestinal inflammation, which may result in intestinal diseases in humans such as inflammatory bowel diseases^7,8^.

Traditionally, apoptosis and necrosis have been thought of as two main types of cell death that are essential for epithelial turnover and tissue homeostasis in the intestine^7^. Recently, necroptosis, a new type of caspase-independent programmed cell death, has been identified in intestinal epithelium, challenging previous concepts^8,9^. Necroptosis has similar morphological characteristics with necrosis, but as apoptosis, is strictly regulated by an intracellular protein platform^10,11^. At present, the most informative studies of necroptosis signaling pathway derive from the tumor necrosis factor (TNF) -triggered system^11^. Briefly, during necroptosis, receptor-interacting protein kinase 1 (RIP1) interacts with RIP3 through RIP homotypic interaction motif, resulting in activation and auto-phosphorylation of RIP3 to form the RIP1/3 containing signaling complex (termed the necrosome)^11,12^. RIP3 binds to and phosphorylates its substrate mixed-lineage kinase domain-like protein (MLKL), and phosphorylated MLKL undergoes oligomerization and membrane translocation to induce membrane depolarization and cell rupture and necrosis^11^. In addition, the RIP1/RIP3 necrosome has been proposed to activate phosphoglycerate mutase family member 5 (PGAM5), and PGAM5 recruits the mitochondrial fission factor dynamin-related protein 1 (DRP1) and activates its GTPase activity by dephosphorylating the serine site of DRP1. DRP1 activation causes mitochondrial fragmentation and reactive oxygen species (ROS) generation, which finally leads to cell necroptosis^13^. The cell rupture and necrosis result in the release of intracellular damage-associated molecular patterns (DAMP) such as high-mobility group box 1 (HMGB1) protein and promotes ongoing inflammation and secondary tissue injury^14^.

Emerging evidence has shown that necroptosis plays an important role in various physiological and pathological disorders^11,15–19^. However, the role of necroptosis in sepsis-induced intestinal injury has been rarely evaluated. In this study, we used a piglet model, a well-characterized animal model for studying human intestinal physiology^20,21^. *Escherichia coli* LPS, a potent inflammatory component of the outer membrane of Gram-negative bacteria, was injected to build the model of intestinal injury during sepsis^22^. We firstly investigated the presence of necroptosis in intestinal injury. The contribution of necroptosis to intestinal injury was also investigated by the use of necrostatin-1 (Nec-1), a well-described inhibitor of necroptosis^23^.

## Materials and methods

### Experimental animals and design

All experiments were approved by the Animal Care and Use Committee of Wuhan Polytechnic University (Wuhan, China). Seventy weanling pigs (male, Duroc × Large White × Landrace, 28 ± 3 d, average body weight 7.1 ± 0.9 kg BW) were purchased from Aodeng Agriculture and Animal Husbandry Technology Co., Ltd (Hubei, China). The piglets were individually housed in an environmentally controlled physiology room and fed a conventional weaned pig diet for 14 days before experiments were initiated. The pigs were allowed *ad libitum* access to feed and water.

In the first experiment, 42 weaned pigs were randomly assigned to seven groups (*n* = 6 pigs/group) including control group and LPS groups slaughtered at six different times. The pigs in LPS groups were injected intraperitoneally with *Escherichia coli* LPS (*Escherichia coli* serotype 055: B5, Sigma Chemical Inc., St. Louis, MO, USA) at 100 μg/kg body weight (BW), and then were sacrificed at 1, 2, 4, 8, 12, or 24 h after LPS challenge. The pigs in control group were sacrificed at 0 h after injection with an equal volume of 0.9% (wt/vol) NaCl solution. The LPS dose was used according to our previous study^22^.

In the second experiment, 28 weaned pigs were randomly allotted into four treatments (*n* = 7 pigs/treatment) in a 2 × 2 factorial experiment. The pigs were pretreated intraperitoneally with necrostatin-1 (Nec-1, MedChem Express, NJ, USA) at 1.0 mg/kg BW or equal volume of 2% DMSO solution 30 min before the intraperitoneal injection of LPS or saline, and the pigs were sacrificed at 4 h after LPS or saline injection. The dose of Nec-1 was chosen in accordance with Koudstaal et al.^24^ and our preliminary experiment in pigs (data not shown).

### Blood and intestinal sample collections

At the time of sacrifice, blood samples were collected into 10-ml uncoated vacuum tube, and centrifuged to separate serum. Sera were stored at −80 °C for further analysis. Another 2 mL blood sample was collected into an EDTA-K3 anticoagulative vacuum tube from the same pig for white blood cell count. Following blood collection, the pigs were humanely euthanized with sodium pentobarbital (80 mg/kg BW). Intestinal segments (3 cm, 1 cm, and 10 cm) were harvested from the mid-jejunum. The 3-cm segments were flushed with ice-cold phosphate-buffered saline, and then placed in 4% paraformaldehyde for morphological analysis. The 1-cm segments were flushed, and then fixed with 2.5% glutaraldehyde for ultrastructure analysis. The 10-cm intestinal segments were opened longitudinally and the contents were flushed. Intestinal mucosa samples were collected with a sterile glass slide, snap-frozen in liquid nitrogen, and then stored at −80 °C for further analysis.

### Blood biomarkers

White blood cell count was assessed by an automated hematology analyzer Sysmex K4500 (TOA Medical Electronics Co., Kobe, Japan). The levels of serum C-reactive protein (CRP), procalcitonin (PCT), D-lactate, and intestinal fatty acid-binding protein (I-FABP) were measured using commercially available porcine ELISA kits (MSKBIO, Wuhan, China).

### Intestinal morphology

After a 24 h fixation, jejunal segments were dehydrated, embedded, sectioned, and stained with hematoxylin and eosin. Villus height and crypt depth were measured according to the methods described in our previous study^22^.

### Intestinal ultrastructure

Jejunal samples were fixed with 2.5% glutaraldehyde, and postfixed in 1% osmium tetroxide. The intestinal samples were then dehydrated with graded concentrations of ethanol, and then embedded in Epon 812 (Eimicon, Shanghai, China). Ultra-thin sections were cut and stained with uranyl acetate and lead citrate. Ultrastructural observation of the jejunum was conducted using a transmission electron microscope (TEM) (Tecnai, FEI, Hillsboro, USA) at an accelerating voltage of 200 kV and a magnification of 5000 in a blind manner.

### Intestinal mucosal disaccharidase activities

Disaccharidase activities in the supernatant of jejunal mucosa were determined using glucose kits (Nanjing Jiancheng Bioengineering Institute, Nanjing, China). Enzyme activity is expressed as U/mg protein.

### Serum and intestinal mucosal pro-inflammatory cytokine concentrations

TNF-α, IL-1β, and IL-6 concentrations in serum and jejunal mucosa supernatant were assayed according to Wang et al.^25^ and the results of jejunal mucosa supernatant were expressed as pg/mg protein. Serum HMGB1 concentration was determined using porcine ELISA kit (Cusabio Technology LLC).

### Protein expression analysis by Western blot

Total proteins and nuclear and cytoplasmic proteins were extracted from jejunal mucosa using a commercially available total protein extraction kit (#KGP250/KGP2100, KeyGEN BioTECH, Jiangsu, China) and a commercially available nuclear and cytoplasmic protein extraction kit (#KGP150/KGP1100, KeyGEN BioTECH, Jiangsu, China), respectively. According to the manufacturer’s instructions, jejunal mucosa samples were suspended in lysis buffer, homogenized, and centrifuged to collect the supernatants. Protein concentrations of the supernatants were quantified by a bicinchoninic acid assay. Nuclear and cytoplasmic protein extracts were used to analyze HMGB1 expression, and total protein extracts were used to analyze other protein expressions. The method for protein expression analysis was in accordance with the procedures of Liu et al.^22^ Specific primary antibodies included rabbit anti-claudin-1 (#51-9000, Invitrogen), mouse anti-occludin (#ab31721, Abcam), rabbit anti-RIP1 (#LS-B8214, LifeSpan), rabbit anti-RIP3 (#SC-135170, Santa Cruz), rabbit anti-total MLKL (t-MLKL) (#37705, Cell Signaling), rabbit anti-phosphorylated MLKL (p-MLKL) (#62233, Cell Signaling), rabbit anti-PGAM5 (*#*ab131552, Abcam), rabbit anti-DRP1 (*#*ab154879, Abcam), rabbit anti-HMGB1 (#PAB12414, Abnova), and mouse anti-β-actin (#A2228, Sigma Aldrich). Phosphorylated form of MLKL was normalized with its abundance, i.e., the total protein content of MLKL. The relative protein abundance of other target proteins were expressed as the ratio of target protein/β-actin protein.

### Immunohistochemistry

Immunohistochemical staining was carried out according to the methods described by Li et al.^26^. Specific primary antibodies against claudin-1, occludin, RIP1, and HMGB1 were the same as those used in Western blot. Specific primary antibodies against CD163 (a cell surface marker of macrophage) (#GB13340) and CD11b (a cell surface marker for monocytes) (#GB11058) were purchased from Servicebio. Images were taken using a microscope (Eclipse Ci-L, Nikon, Tokyo, Japan) and analyzed by Image-Pro Plus 6.0 software (Media Cybemetics, MD, USA).

### mRNA expression analysis by real-time PCR

RNA isolation, quantification, reverse transcription, and real-time PCR were performed according to the methods described by Liu et al.^22^. The primer pairs used are shown in Supplemental Table 1 to amplify the target genes. The mRNA expression of the target genes relative to housekeeping gene (β-actin) was calculated by the 2^-△△CT^ method^27^.

### Statistical analysis

In the first experiment, the data were analyzed using Student’s t-test. In the second experiment, the data were analyzed by ANOVA using the general linear model procedures of Statistical Analysis System (SAS Inst. Inc., Cary, NC, USA). Post hoc testing was conducted using Duncan’s multiple comparison tests. All data were presented as means with standard errors of means. *P* ≤ 0.05 was considered as statistically significant.

## Results

### LPS induces dynamical changes of blood biomarkers for diagnosis of sepsis in piglets

**Fig. 1:**
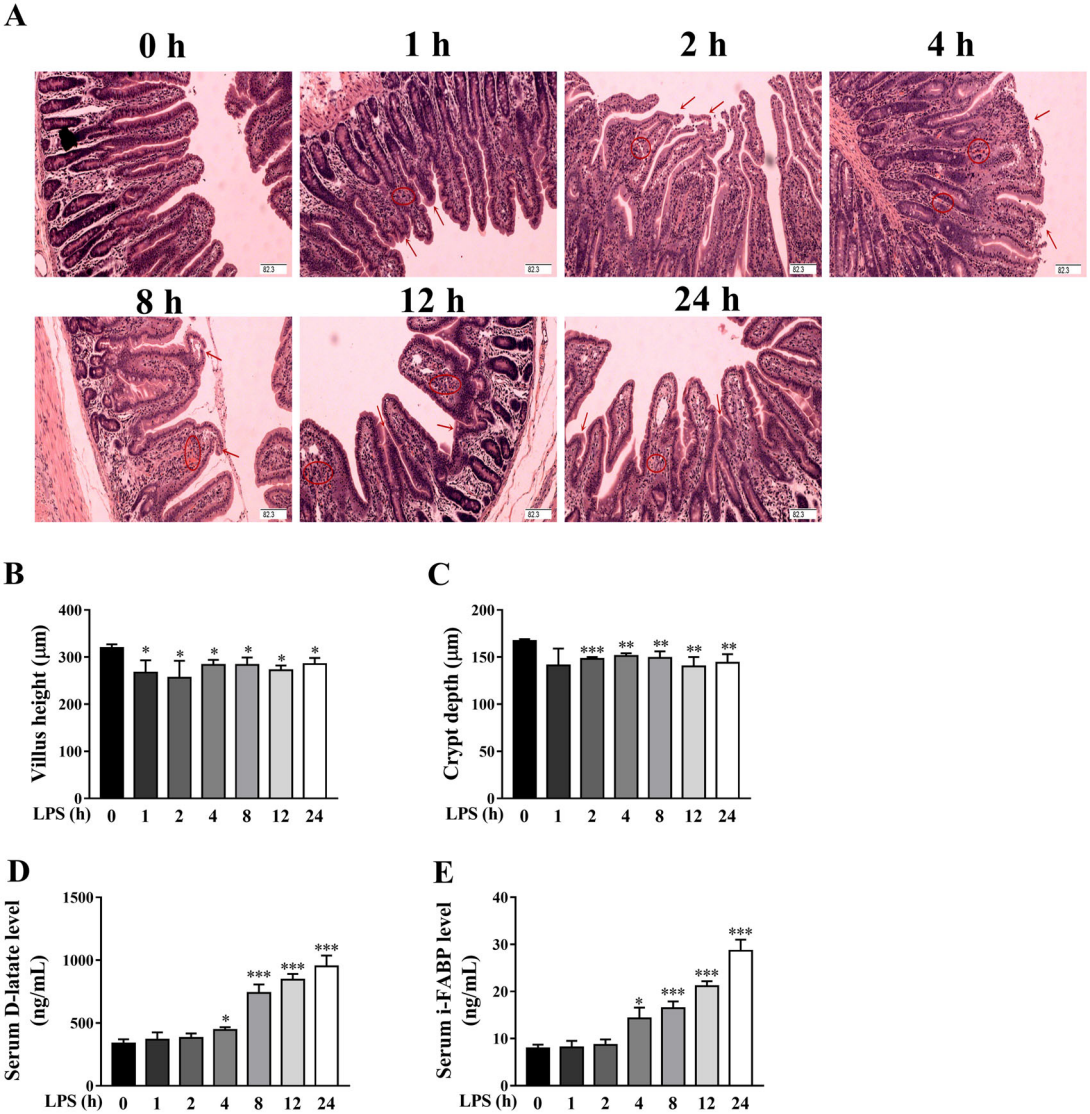
LPS induces dynamically intestinal injury in piglets. **A** Jejunal morphological characteristics (hematoxylin and eosin-stained). Arrows indicate lifting of epithelium at the tip of the villus, and villous atrophy. Circles indicate hemorrhage in the lamina propria. Original magnification: 100×, scale bars = 82.3 μm. **B**, **C** Jejunal villus height and crypt depth. **D** Serum D-lactate level. **E** Serum intestinal fatty acid binding protein (I-FABP) level. The pigs in control group were sacrificed at 0 h after injection with NaCl solution. The pigs in LPS groups were injected with LPS at 100 μg/kg body weight, and then were killed at 1, 2, 4, 8, 12, or 24 h after LPS challenge. Values are means ± SE, *n* = 6. ***p < 0.001, **p < 0.01 and *p < 0.05, significantly different from the control group (0 h).

To demonstrate the clinical relevance of LPS model with respect to sepsis, we measured total white blood cell count, and serum CRP and PCT levels, several typical blood biomarkers for diagnosis of sepsis^28,29^, in piglets at different time points (1, 2, 4, 8, 12, and 24 h) after LPS challenge. Compared to the control group (0 h), total white blood cell count was significantly decreased at 1-4 h (*p* < 0.05), and returned to the normal level at 8 h, and then was increased at 12-24 h (*p* < 0.05) (Fig. 1S). Serum CRP and PCT levels were significantly increased at 4-24 h (*p* < 0.05) (Fig. 1S).

### LPS induces dynamically intestinal injury and dysfunction in piglets

To explore the dynamic effect of LPS challenge on intestinal morphology, we measured jejunal morphologic changes of piglets. Compared to control group, LPS, at different time points, induced different degrees of intestinal morphologic changes demonstrated as lifting of epithelium at the tip of the villus, villous atrophy and hemorrhage in the lamina propria (Fig. 1A). Further analysis showed that LPS led to decreased villus height from 1 to 24 h (Fig. 1B) and crypt depth from 2 to 24 h (Fig. 1C) (*p* < 0.05). We also measured serum D-lactate and I-FABP levels, the typical blood biomarkers for diagnosis of intestinal injury^30,31^. Compared to control group, LPS challenge increased serum D-lactate (Fig. 1D) and I-FABP (Fig. 1E) levels at 4-24 h (*p* < 0.05).

**Fig. 2:**
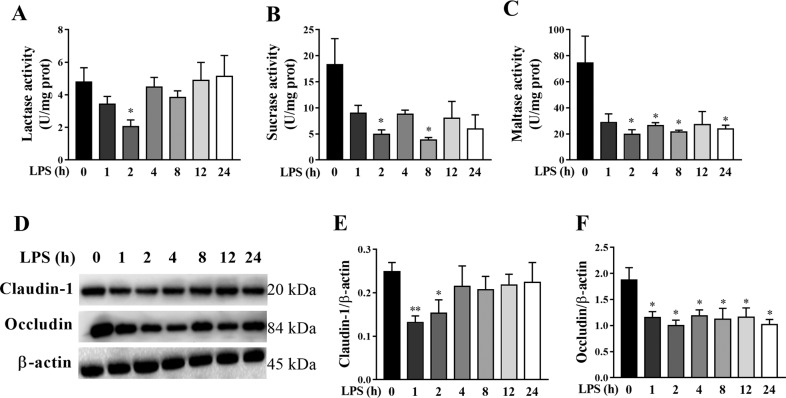
LPS induces dynamically intestinal digestive and barrier dysfunction in piglets. **A**–**C** disaccharidase activities. **D**–**F** Protein expression of claudin-1 and occludin. The pigs in the control group were sacrificed at 0 h after injection with NaCl solution. The pigs in LPS groups were injected with LPS at 100 μg/kg body weight, and then were killed at 1, 2, 4, 8, 12, or 24 h after LPS challenge. Values are means ± SE, *n* = 6. **p < 0.01 and *p < 0.05, significantly different from the control group (0 h).

To investigate the dynamic effect of LPS challenge on intestinal digestive function, we assessed jejunal disaccharidase activities in piglets. Compared to control group, LPS challenge decreased lactase activity (Fig. 2A) at 2 h, sucrase activity (Fig. 2B) at 2 and 8 h, and maltase activity (Fig. 2C) at 2, 4, 8, and 24 h after LPS challenge (*p* < 0.05).

We also measured the protein expression of tight junction proteins claudin-1 and occludin in jejunum of piglets (Fig. 2D–F). Compared to control group, LPS challenge resulted in a significant decrease in protein expression of claudin-1 (Fig. 2E) at 1–2 h, and occludin (Fig. 2F) at all time points after LPS challenge (*p* < 0.05).

### LPS induces dynamically intestinal inflammatory response in piglets

**Fig. 3:**
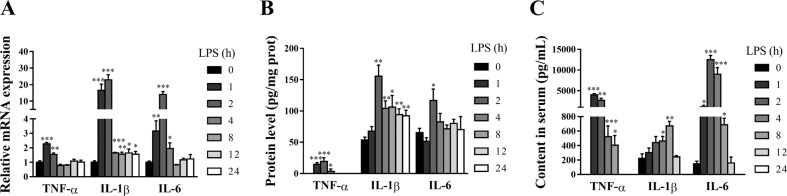
LPS induces dynamically intestinal inflammatory response in piglets. **A** mRNA expression of jejunal TNF-α, IL-1β, and IL-6. **B** Protein expression of jejunal TNF-α, IL-1β, and IL-6. **C** Concentrations of serum TNF-α, IL-1β, and IL-6. The pigs in control group were killed at 0 h after injection with NaCl solution. The pigs in LPS groups were injected with LPS at 100 μg/kg body weight, and then were sacrificed at 1, 2, 4, 8, 12, or 24 h after LPS challenge. Values are means ± SE, *n* = 6. ***p < 0.001, **p < 0.01 and *p < 0.05, significantly different from the control group (0 h).

Intestinal injury is closely associated with intestinal inflammation^22^. To assess the dynamic effect of LPS on intestinal inflammatory response of pigs, mRNA and protein expression of jejunal TNF-α, IL-1β, and IL-6, and serum concentrations of TNF-α, IL-1β, and IL-6 were analyzed (Fig. 3A–C). LPS caused a significant, transient induction in these pro-inflammatory cytokines. Specifically, TNF-α mRNA was increased by 2.27 fold at 1 h, and 1.54 at 2 h (*p* < 0.01), and declined to the basal level after 4 h. IL-1β and IL-6 mRNA were increased at 1 h (16.59 and 3.15 fold), and peaked at 2 h (22.87 and 14.03 fold) (*p* < 0.01). The LPS-induced increase of IL-6 mRNA returned to the basal level from 8 h. However, IL-1β mRNA still maintained slight increase at 4–24 h (*p* < 0.05). Similarly, LPS-induced increased protein expression of TNF-α at 1–4 h (maximal at 2 h), IL-1β at 2–24 h (maximal at 2 h), and IL-6 at 2 h (*p* < 0.05). LPS also increased serum concentrations of TNF-α at 1–8 h (maximal at 1 h), IL-1β at 4–8 h (maximal at 8 h) and IL-6 at 1–8 h (maximal at 2 h) (*p* < 0.05).

### LPS induces dynamically intestinal cell necroptosis in piglets

**Fig. 4:**
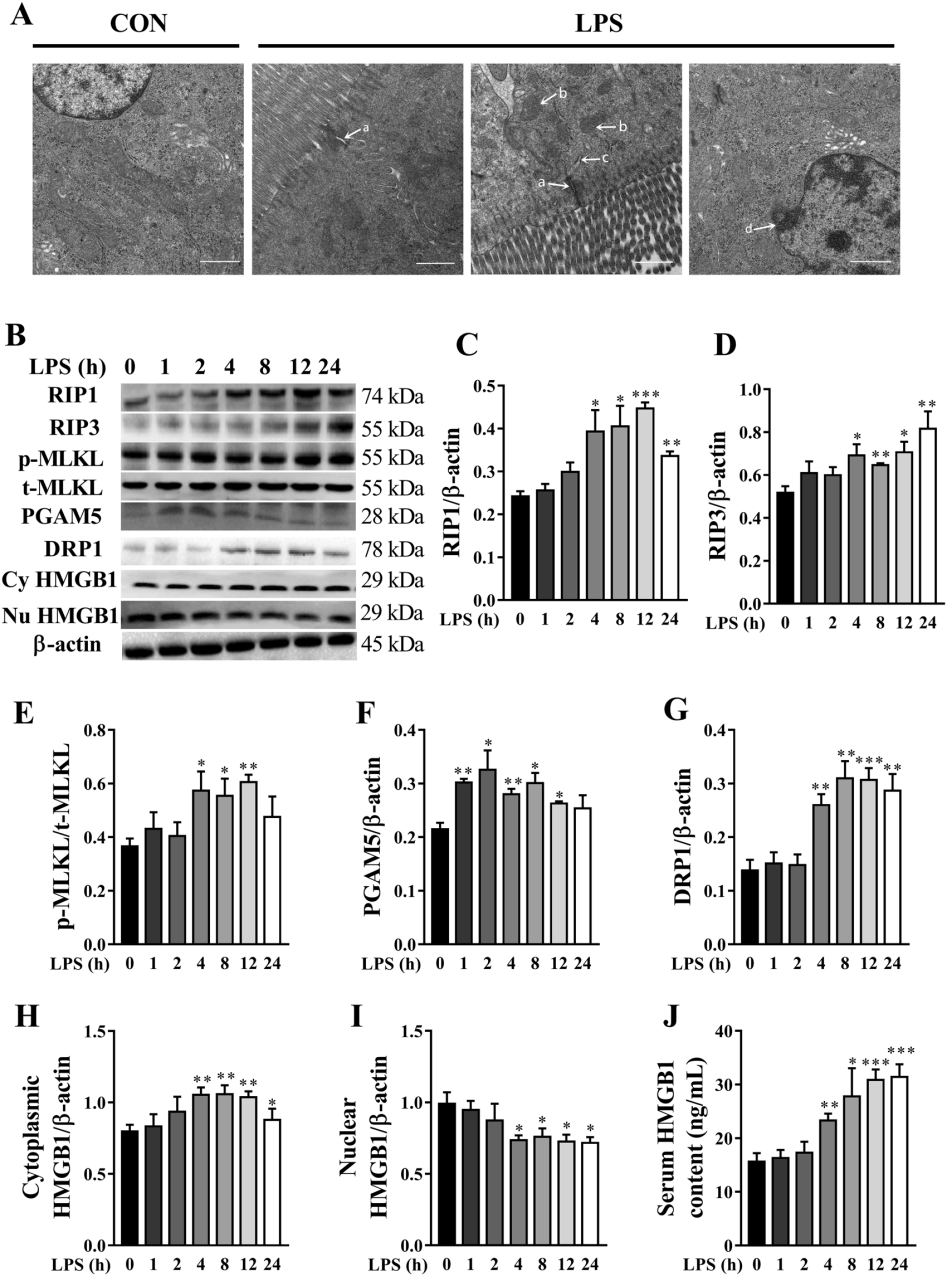
LPS induces dynamically intestinal cell necroptosis in jejunum of piglets. **A** The representative jejunal ultrastructural images from control (Con) group and LPS groups. Compared to Con group, ultrastructural injury including intercellular space enlargement **A**, mitochondria swelling and cristae disappearance **B**, endoplasmic reticulum expansion (**C**) as well as nuclear deformation, nuclear membrane rupture, and chromatin overflowing (**D**) were observed in LPS groups. Original magnification: ×5000, scale bars = 1 μm. **B**–**J** Protein expression of necroptosis-related signals, and serum HMGB1 content. Cy HMGB1, cytoplasmic HMGB1. Nu HMGB1, nuclear HMGB1. The pigs in the control group were sacrificed at 0 h after injection with NaCl solution. The pigs in LPS groups were injected with LPS at 100 μg/kg body weight, and then were sacrificed at 1, 2, 4, 8, 12, or 24 h after LPS challenge. Values are means ± SE, *n* = 6. ***p < 0.001, **p < 0.01 and *p < 0.05, significantly different from the control group (0 h).

Necroptosis has been considered as a new form of cell death, which is closely associated with tissue injury and inflammation^8^. To investigate the presence of necroptosis in the intestine of LPS-challenged pigs, we examine ultrastructure of jejunum by TEM (Fig. 4A). Compared to control group, ultrastructure injury including intercellular space enlargement, endoplasmic reticulum expansion, mitochondria swelling and cristae disappearance as well as nuclear deformation, nuclear membrane rupture, and chromatin overflowing were observed in jejunum in LPS-challenged pigs.

To further investigate the presence of necroptosis in the intestine of LPS-challenged pigs, we determined the dynamic change of the protein expression of RIP1, RIP3, and MLKL, which are the key components to drive the necroptosis^8^. Compared to control group, LPS-induced a time-dependent increase in protein expression of RIP1, RIP3, and phosphorylated MLKL in jejunum of pigs (Fig. 4B–E). Specifically, RIP1 expression was increased at 4–24 h, and was maximal at 12 h (85% higher) (*p* < 0.05). RIP3 expression was increased at 4–24 h, and highest at 24 h (58% higher) (*p* < 0.05). p-MLKL expression was increased at 4–12 h, and peaked at 12 h (66% higher) (*p* < 0.05).

We further measured the mitochondrial proteins PGAM5 and DRP1 that are important for necroptosis execution^13^. Compared to control group, LPS led to a time-dependent increase in protein expression of PGAM5 and DRP1 in jejunum of pigs (Fig. 4F, G). Specifically, PGAM5 expression was increased at 1-12 h, and was maximal at 2 h (52% higher) (*p* < 0.05). DRP1 expression was increased at 4-24 h, and peaked at 8 h (125% higher) (*p* < 0.05). In addition, HMGB1, a representative DAMP released by necrotic cells, was determined (Fig. 4H–J). We found that higher amounts of HMGB1 were translocated from the nucleus to the cytoplasm, and then were released to the serum. Specifically, cytoplasmic and serum HMGB1 amounts were increased at 4-24 h (maximal at 8 and 24 h, respectively), and nuclear HMGB1 amount was decreased at 4-24 h (minimum at 24 h).

### Nec-1 inhibits intestinal cell necroptosis in LPS-challenged piglets

**Fig. 5:**
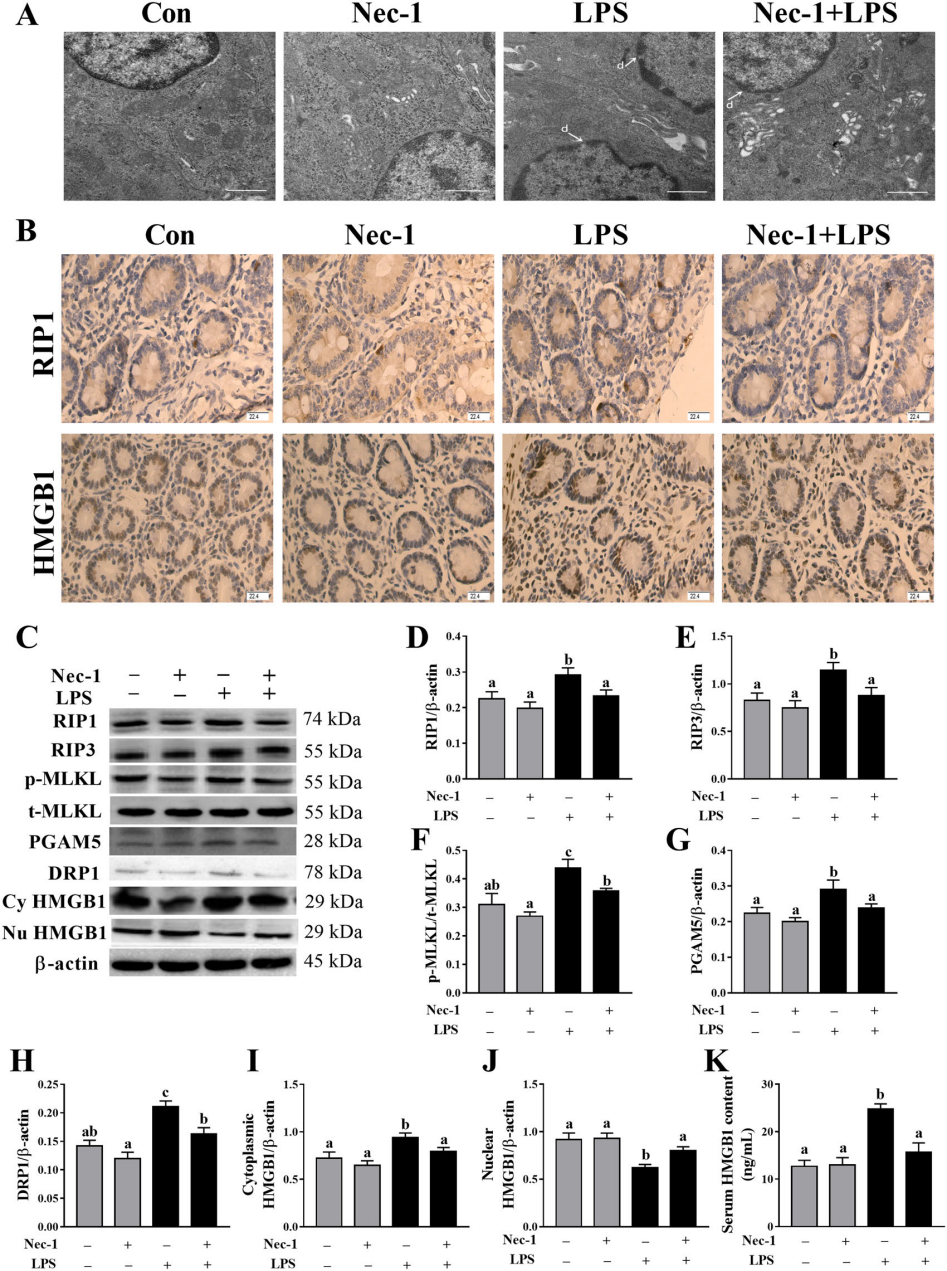
Nec-1 pretreatment inhibits intestinal cell necroptosis in LPS-challenged piglets. **A** The representative jejunal ultrastructural images by TEM. LPS-induced necrotic ultrastructural changes such as nuclear deformation, nuclear membrane rupture and chromatin overflowing **D**, and Nec-1 reduced the necrotic ultrastructural changes. Original magnification: 5000 ×, scale bars = 1 μm. **B** Localization of RIP1 and HMGB1 by immunohistochemistry staining. Original magnification: 400 ×, scale bars = 22.4 μm. **C**–**K** Protein expression of necroptosis-related signals by western blotting, and serum HMGB1 content by ELISA. Cy HMGB1, cytoplasmic HMGB1. Nu HMGB1, nuclear HMGB1. The pigs were pretreated intraperitoneally with Nec-1 at 1.0 mg/kg body weight or equal volume of 2% DMSO solution 30 min before the intraperitoneal injection of LPS or saline, and the pigs were sacrificed at 4 h after LPS or saline injection. Values are means ± SE, n = 7. ^abc^Means without a common letter differ, *p* < 0.05.

To further demonstrate that LPS-induced intestinal injury observed above is partially due to the contribution of necroptosis, 30 min before LPS challenge, the pigs were pretreated with Nec-1, an inhibitor of RIP1 kinase activity^23^ and RIP1/RIP3 association^12^. Firstly, ultrastructural analysis showed that Nec-1 reduced the necrotic ultrastructural changes in jejunum (Fig. 5A). In addition, immunohistochemistry staining showed that RIP1 and HMGB1 were induced in intestinal gland epithelium cells at 4 h after LPS challenge but were inhibited after Nec-1 pretreatment (Fig. 5B). Consistently, Nec-1 inhibited protein expression of RIP1, RIP3 and phosphorylated MLKL as well as PGAM5 and DRP1 (*p* < 0.05) (Fig. 5C–H). Nec-1 also alleviated the translocation of HMGB1 from the nucleus to the cytoplasm, and the release of HMGB1 to the serum (Fig. 5I–K).

### Inhibition of necroptosis by Nec-1 attenuates LPS-induced sepsis in piglets

We explored if nec-1 pretreatment could attenuate sepsis-induced by LPS challenge. LPS challenge at 4 h significantly decreased total white blood cell count, and increased serum CRP and PCT levels (*p* < 0.05) (Fig. 2S). However, Nec-1 pretreatment attenuated the decrease of total white blood cell count, and the increase of serum CRP and PCT levels by LPS challenge (*p* < 0.05).

### Inhibition of necroptosis by Nec-1 protects from intestinal injury and dysfunction in LPS-challenged piglets

**Fig. 6:**
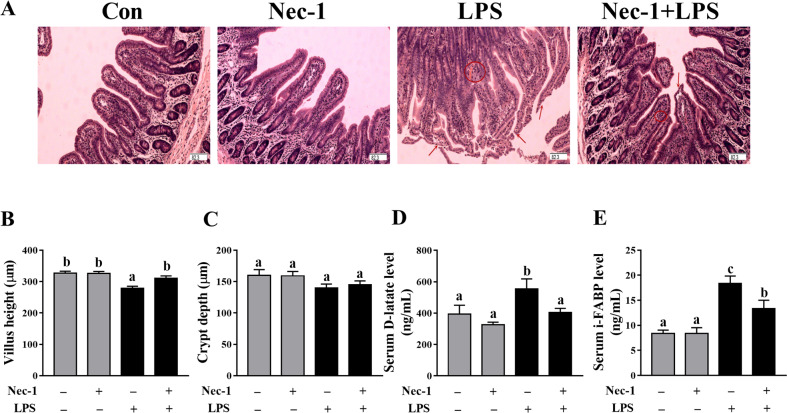
Inhibition of necroptosis by Nec-1 protects from intestinal injury in LPS-challenged piglets. **A** Jejunal morphological characteristics (haematoxylin and eosin stained). Arrows indicate lifting of epithelium at the tip of the villus, and villous atrophy. Circles indicate hemorrhage in the lamina propria. Original magnification: ×100, scale bars = 82.3 μm. **B**, **C** Jejunal villus height and crypt depth. **D** Serum D-lactate level. **E** Serum intestinal fatty acid-binding protein (I-FABP) level. The pigs were pretreated intraperitoneally with Nec-1 at 1.0 mg/kg body weight or equal volume of 2% DMSO solution 30 min before the intraperitoneal injection of LPS or saline, and the pigs were sacrificed at 4 h after LPS or saline injection. Values are means ± SE, *n* = 7. ^abc^Means without a common letter differ, *p* < 0.05.

We investigated if nec-1 pretreatment could attenuate LPS-induced intestinal injury (Fig. 6A) LPS challenge at 4  h significantly decreased villus height in jejunum of piglets (*p*   < 0.05) (Fig. 6B, C). However, inhibition of necroptosis by Nec-1 reversed the decrease of villus height induced by LPS (*p*  <  0.05). Nec-1 treatment had no effect on crypt depth. LPS challenge at 4 h increased serum D-lactate and I-FABP levels (Fig. 6D, E). However, inhibition of necroptosis by Nec-1 alleviated the increase of serum D-lactate and I-FABP levels induced by LPS (*p*   < 0.05).

**Fig. 7:**
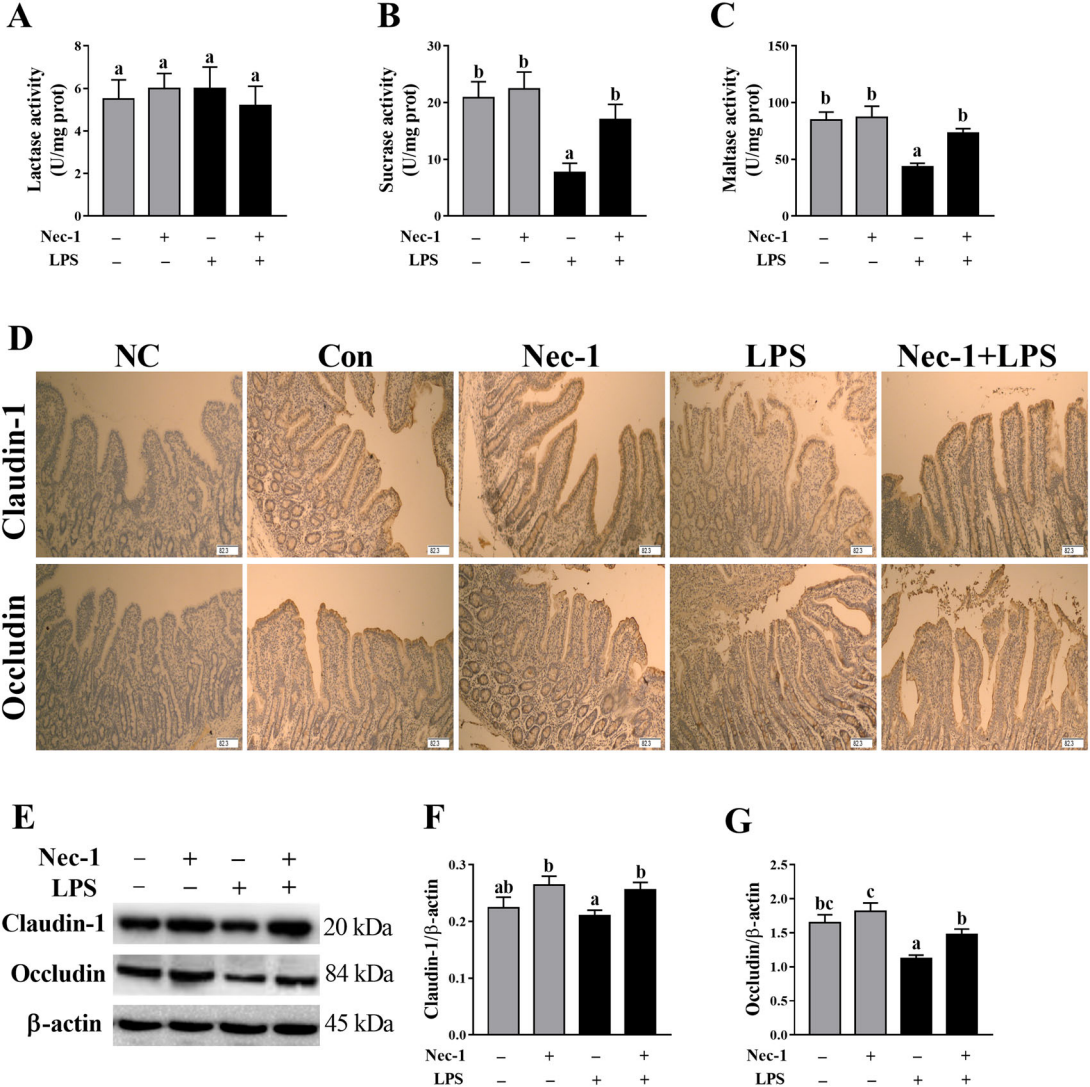
Inhibition of necroptosis by Nec-1 protects from intestinal digestive and barrier dysfunction in LPS-challenged piglets. **A**–**C** disaccharidase activities. **D** Localization of claudin-1 and occludin by immunohistochemistry staining. Original magnification: 100 ×, scale bars = 82.3 μm. **E**–**G** Protein expression of claudin-1 and occludin by western blotting. The pigs were pretreated intraperitoneally with Nec-1 at 1.0 mg/kg body weight or equal volume of 2% DMSO solution 30 min before the intraperitoneal injection of LPS or saline, and the pigs were sacrificed at 4 h after LPS or saline injection. Values are means ± SE, *n* = 7. ^abc^Means without a common letter differ, *p* < 0.05.

We measured intestinal disaccharidase activities to explore whether Nec-1 pretreatment could attenuate the injury of intestinal digestive function. LPS challenge at 4 h significantly reduced the activities of jejunal sucrase and maltase (*p* < 0.05) (Fig. 7A–C). Inhibition of necroptosis with Nec-1 attenuated LPS-induced decrease of jejunal sucrase and maltase activities in piglets (*p* < 0.05).

Protein expression of claudin-1 and occludin was determined by immunohistochemistry and western blotting to investigate whether nec-1 pretreatment could attenuate the injury of intestinal barrier function (Fig. 7D–G). Western blotting analysis showed that LPS challenge at 4 h significantly down-regulated the protein expression of jejunal occludin (*p* < 0.05). Inhibition of necroptosis with Nec-1 enhanced protein expression of claudin-1 and occludin (*p* < 0.05), which was also demonstrated by immunohistochemistry analysis.

### Inhibition of necroptosis by Nec-1 decreased intestinal inflammation in LPS–challenged piglets

**Fig. 8:**
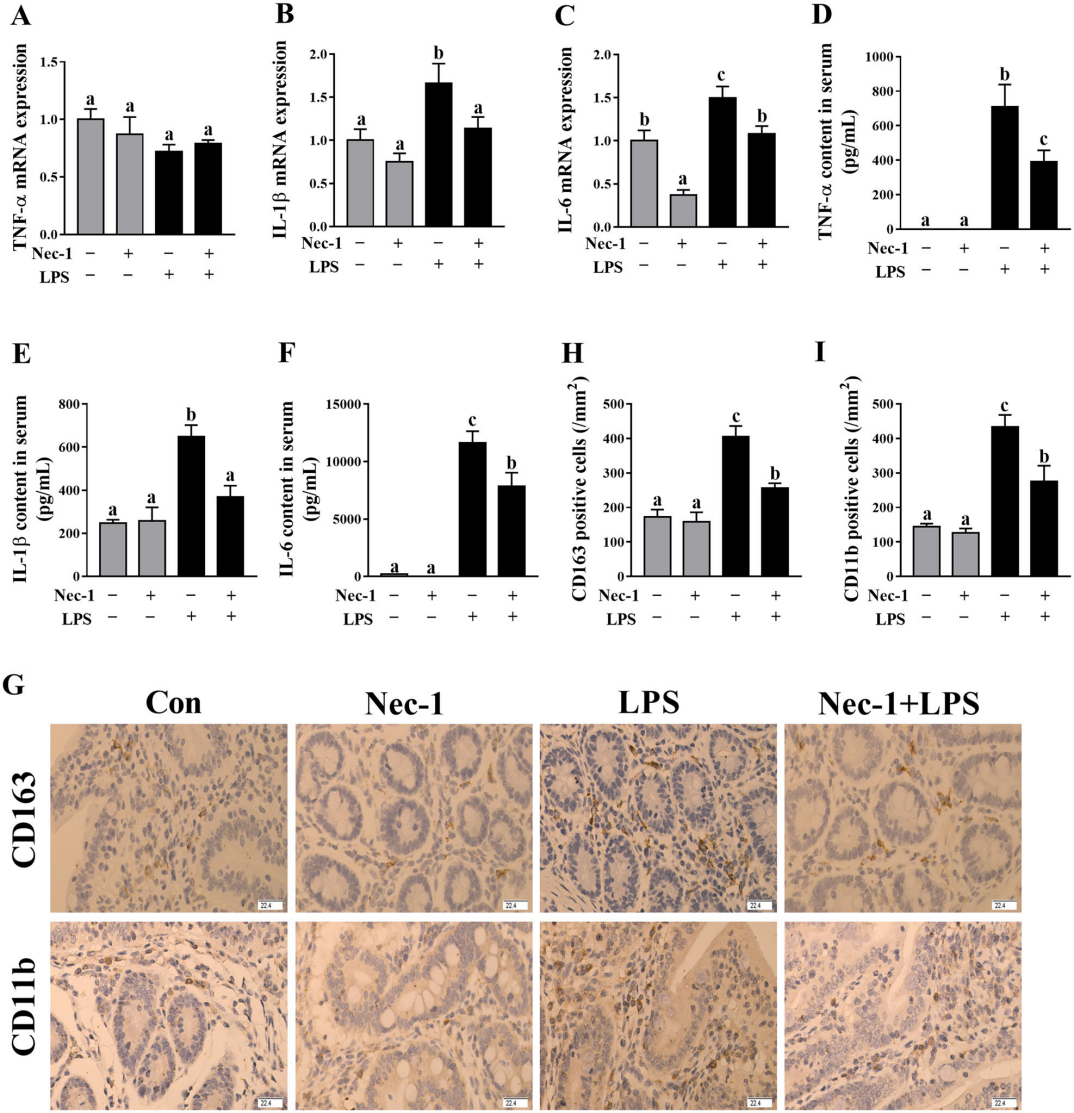
Inhibition of necroptosis by Nec-1 decreased intestinal inflammation in LPS-challenged piglets. **A**–**C** mRNA expression of jejunal TNF-α, IL-1β, and IL-6. **D**–**F** Concentrations of serum TNF-α, IL-1β, and IL-6. **G**–**I** Immunohistochemistry for CD163 and CD11b. The density of positive cells was calculated as the number of positive cells per mm^2^ area. Original magnification: 400 ×, scale bars = 22.4 μm. The pigs were pretreated intraperitoneally with Nec-1 at 1.0 mg/kg body weight or equal volume of 2% DMSO solution 30 min before the intraperitoneal injection of LPS or saline, and the pigs were killed at 4 h after LPS or saline injection. Values are means ± SE, *n* = 7. ^abc^Means without a common letter differ, *p* < 0.05.

To demonstrate that necroptosis contributes to intestinal inflammation, firstly, we determined the mRNA expression of jejunal TNF-α, IL-1β, and IL-6 (Fig. 8A–C) and concentrations of serum TNF-α, IL-1β, and IL-6 (Fig. 8D–F). LPS challenge at 4 h significantly increased the mRNA expression of IL-1β and IL-6 in jejunum, and serum concentrations of TNF-α, IL-1β, and IL-6 (*p* < 0.05). However, inhibition of necroptosis by Nec-1 alleviated the increase of IL-1β and IL-6 mRNA expression in jejunum, and of TNF-α, IL-1β, and IL-6 concentrations in serum (*p* < 0.05). Further, immunohistochemistry for CD163 (a macrophage marker) and CD11b (a monocyte marker) was performed. We found that LPS challenge at 4 h resulted in an increase of CD163 and CD11b positive cell numbers in jejunum, but were inhibited by Nec-1 pretreatment (Fig. 8G–I).

## Discussion

In addition to necrosis and apoptosis, a new type of cell death has been identified and described in recent years, termed necroptosis^8,9,32^. Necroptosis has the same morphological features of dying cells with necrosis. However, in contrast to necrosis, a passive form of cell death, necroptosis is strictly modulated by an intracellular protein platform, similar to apoptosis^10,11^. Emerging evidence has demonstrated that necroptosis plays an important role in a variety of physiological and pathological disorders^11,15–19^. Recently, several studies have shown that necroptosis of intestinal epithelial cells can result in intestinal inflammation or injury in inflammatory bowel disease in human^33^, and intestinal ischemia/reperfusion in rats^34^. However, a thorough understanding about the relationship between necroptosis and intestinal injury during sepsis is limited.

In this study, LPS-induced sepsis was demonstrated in piglets by blood biomarkers for diagnosis of sepsis (total white blood cell count, serum CRP, and PCT). We firstly investigated the dynamic effect of LPS challenge on intestinal injury in a piglet model. Expectedly, we found that, at different time points (1, 2, 4, 8, 12, and 24 h), LPS-induced different degrees of intestinal morphologic changes. Further analysis showed that LPS led to decreased villus height from 1 to 24 h and crypt depth from 2 to 24 h after LPS challenge. These results indicate that LPS caused intestinal morphological damage, which is further demonstrated by blood biomarkers for the diagnosis of intestinal injury (serum D-lactate and I-FABP). Generally, the damage of intestinal structure is often accompanied by disorder of digestion, absorption, and barrier functions. Similarly, we found that LPS challenge resulted in decreased disaccharidase activities at 2–24 h, and protein expression of tight junction proteins claudin-1 at 1–2 h and occludin at 1–24 h. Our data suggest that LPS-induced intestinal digestive and barrier dysfunction. Until now, the research on the dynamic effect of LPS on intestinal structure and function is lacking. The results of our current study are consistent with our previous reports in pigs at a single time point (4, 6, or 24 h) after LPS challenge^22,35,36^.

Intestinal damage is closely related to intestinal inflammation^22,25^. In the present study, LPS caused a significant, transient (1-2 h) induction in jejunal TNF-α, IL-1β, and IL-6. The maximal response for jejunal IL-1β and IL-6 mRNA occurred at approximately 2 h post-injection, and remained slightly elevated from 4 to 24 h and 4–8 h, respectively. However, the maximal response for TNF-α mRNA appeared at approximately 1 h, and began to decline to the basal level thereafter. The maximal response for jejunal TNF-α, IL-1β, and IL-6 protein also occurred at 2 h post-injection. The response for these cytokines in serum was similar to that in jejunum. The ability of LPS to increase these cytokines in the intestine in pigs at a single time point (4 or 6 h) has been previously reported^22,35^. However, there is no research on the dynamic effect of LPS on intestinal pro-inflammatory cytokines. In the present study, the temporal pattern of LPS-induced changes in mRNA and protein abundance for TNF-α, IL-1β, and IL-6 in the intestine is similar to the previous observations in other tissues such as gastrocnemius, heart, liver, spleen, and kidney in rats^37^. The early and transient pattern (1–2 h) of LPS-induced increase in pro-inflammatory cytokines might contribute to the early injury of intestinal structure and function.

We further investigated if LPS-induced intestinal injury was accompanied by necroptosis. Ultrastructure observations of jejunum by TEM showed that LPS-induced mitochondria swelling and indistinct cristae as well as nuclear deformation, nuclear membrane rupture, and chromatin overflowing, indicating obvious intestinal cell necrosis. We further explored the dynamic effect of LPS on necroptosis signaling pathway for the first time. RIP1, RIP3, and MLKL are the key components for necroptosis^11,12^. Their downstream mitochondrial proteins PGAM5 and DRP1 have been shown to be important for necroptosis execution^13^. We observed that RIP1 expression was significantly increased at 4–24 h after LPS challenge, and was maximal at 12 h. RIP3 expression was increased at 4–24 h, and highest at 24 h. MLKL phosphorylation was increased at 4–12 h and peaked at 12 h. In addition, PGAM5, DRP1, and cytoplasmic and serum HMGB1 were increased at 4–24 h. Nowadays, the studies about the effect of LPS on necroptosis in the intestine are lacking. Similar to our data on the intestine, Shao et al.^38^ reported that RIP1 expression was up-regulated in a time-dependent manner and peaked at 12–24 h after LPS treatment in the brain cortex of rats. Our results demonstrated that LPS-induced necroptosis and activated necroptosis signaling pathway at a later period (4–24 h) after LPS challenge, which might contribute to the injury of intestinal structure and function at the later phase of LPS challenge.

To further demonstrate that LPS-induced intestinal injury is partially due to the contribution of necroptosis, we used Nec-1, an inhibitor of RIP1 kinase activity^23^ and RIP1/RIP3 association^12^, to block necroptosis^39^. As expected, pretreatment with Nec-1 reduced the necrotic ultrastructural changes, and decreased protein amounts of RIP1, RIP3, and phosphorylated MLKL as well as PGAM5, DRP1, and cytoplasmic and serum HMGB1, which indicates that Nec-1 was effective to inhibit necroptosis signaling pathway in jejunum of piglets. Pretreatment with Nec-1 significantly attenuated the injury of intestinal morphology and the decrease of disaccharidases activities, and increased protein expression of claudin-1 and occludin in LPS-challenged pigs. Until now, the studies about the effect of inhibition of necroptosis by Nec-1 on in LPS-induced intestinal injury is lacking. Li et al.^40^ reported that targeting necroptosis of intestinal epithelial cells by Nec-1 alleviated intestinal injury after intestinal ischemia/reperfusion in rats. Cui et al.^41^ showed that gut barrier dysfunction induced by aggressive fluid resuscitation in severe acute pancreatitis was alleviated by necroptosis inhibition in rats. Our results demonstrate that the occurrence of necroptosis resulted in LPS-induced impairment of intestinal structure and function in pigs.

We further investigated if necroptosis contributed to intestinal inflammation. We found that inhibition of necroptosis by Nec-1 downregulated mRNA expression of IL-1β and IL-6 in jejunum, and decreased the concentrations of TNF-α, IL-1β, and IL-6 in serum. In addition, Nec-1 pretreatment also alleviated the increase of inflammatory cells such as marcrophages and monocytes in jejunum. In agreement with our findings, Liu et al.^42^ reported that treatment with Nec-1 suppressed excessive production of IL-6 in acute dextran sulfate sodium-induced colitis in mice. Negroni et al.^34^ found that targeting necroptosis through Nec-1 reduced intestinal inflammation in vitro and in cultured intestinal explants from inflammatory bowel diseases. Our study suggests that the occurrence of necroptosis enhanced inflammation in the intestine of LPS-challenge pigs. According to our data, we demonstrate that necroptosis is a later event (4–24 h) that occurs when intestinal inflammation has already been established (1–2 h). However, once necroptosis is triggered, it actively take part in determining the degree of the inflammation itself.

There were several limitations in our study. First, Nec-1 was applied before the sepsis was induced (LPS challenge), indicating that Nec-1 may have a preventive effect on intestinal injury during sepsis. However, pretreatment with Nec-1 has no clinically relevant compared to posttreatment with Nec-1. So, it is not sure if Nec-1 is able to reduce the already established sepsis in clinical patients. Further research is needed to determine practical and effective clinical use regimens of Nec-1 after sepsis. Second, besides being the necroptosis inhibitor targeting RIP1, Nec-1 is also the inhibitor of indoleamine 2,3-dioxygenase (IDO)^43^. Some literature has shown that blockade of IDO protected against LPS-induced endotoxin shock, and reduced mortality from peritonitis and sepsis in mice^44,45^. Thus, it is difficult to distinguish between the impact of necroptosis and IDO in this study. Further experiments are needed to rule out the potential effects of IDO by using necrostatin 1 s (Nec-1s, a stable variant of necrostatin-1), which is a highly specific necroptosis inhibitor selectively targeting RIP1 but not IDO^46^.

In conclusion, our study demonstrates, for the first time, that LPS induces necroptosis and activates necroptosis signaling pathway in the intestine, which is accompanied by the impairment of intestinal morphology and function. Inhibition of necroptosis with Nec-1 attenuates LPS-induced injury of intestinal morphology and function. Therefore, it is suggested that necroptosis is present and contributes to LPS-induced intestinal injury. Nec-1 may have a preventive effect on intestinal injury during sepsis.

## Supplementary information

Supplementary Table 1

Supplementary Figure 1

Supplementary Figure 2

Supplementary Figure Legends
