# Supplementary material for: Necroptosis is active and contributes to intestinal injury in a piglet model with lipopolysaccharide challenge
Source: Cell Death Dis. 2021 Jan 11;12(1):62. doi: 10.1038/s41419-020-03365-1 (PMC7801412; doi:10.1038/s41419-020-03365-1)
Supplement: Supplementary file 1 — Supplementary Table 1 [file 41419_2020_3365_MOESM1_ESM.docx]

**Supplementary table 1. Primer sequences used for real-time PCR**

| Gene | Forward (5'-3') | Reverse (5'-3') |
| --- | --- | --- |
| TNF-α | AAGACACCATGAGCACTGAGA | CGACCAGGAGGAAGGAGAAG |
| IL-1β | GCTAACTACGGTGACAACAATAATG | CTTCTCCACTGCCACGATGA |
| IL-6 | AAGGTGATGCCACCTCAGAC | TCTGCCAGTACCTCCTTGCT |
| β-actin | TGCGGGACATCAAGGAGAAG | AGTTGAAGGTGGTCTCGTGG |

IL, interleukin; TNF-α, tumor necrosis factor-α.
