# Supplementary figures and images for: Necroptosis is active and contributes to intestinal injury in a piglet model with lipopolysaccharide challenge
Source: Cell Death Dis. 2021 Jan 11;12(1):62. doi: 10.1038/s41419-020-03365-1 (PMC7801412; doi:10.1038/s41419-020-03365-1)

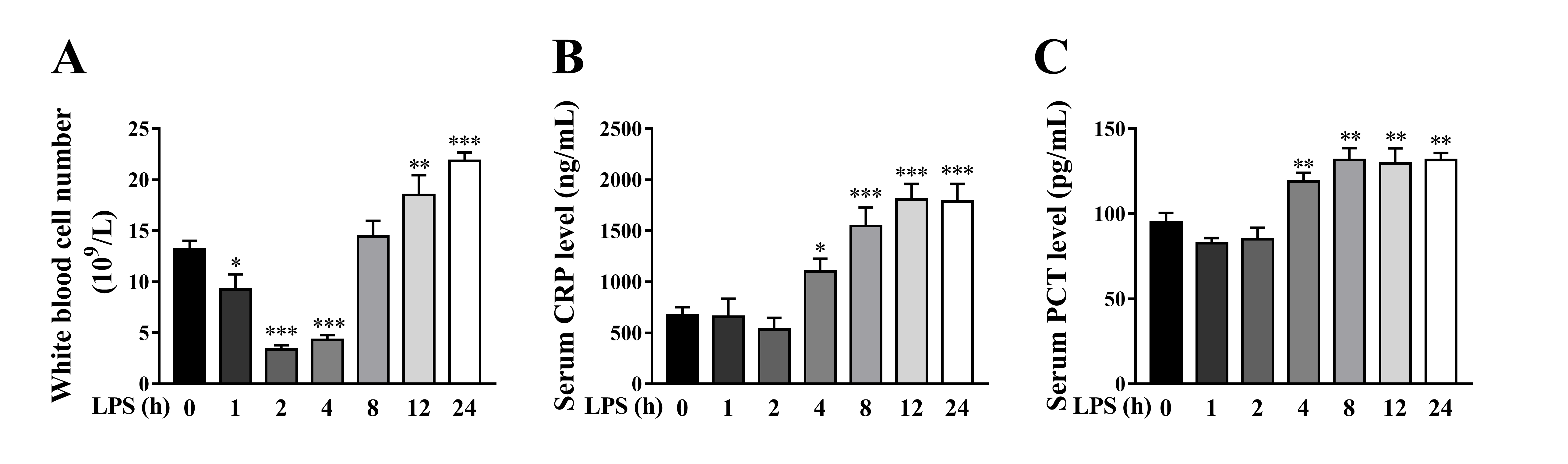

Supplement: Supplementary file 2 — Supplementary Figure 1 [file 41419_2020_3365_MOESM2_ESM.tif]

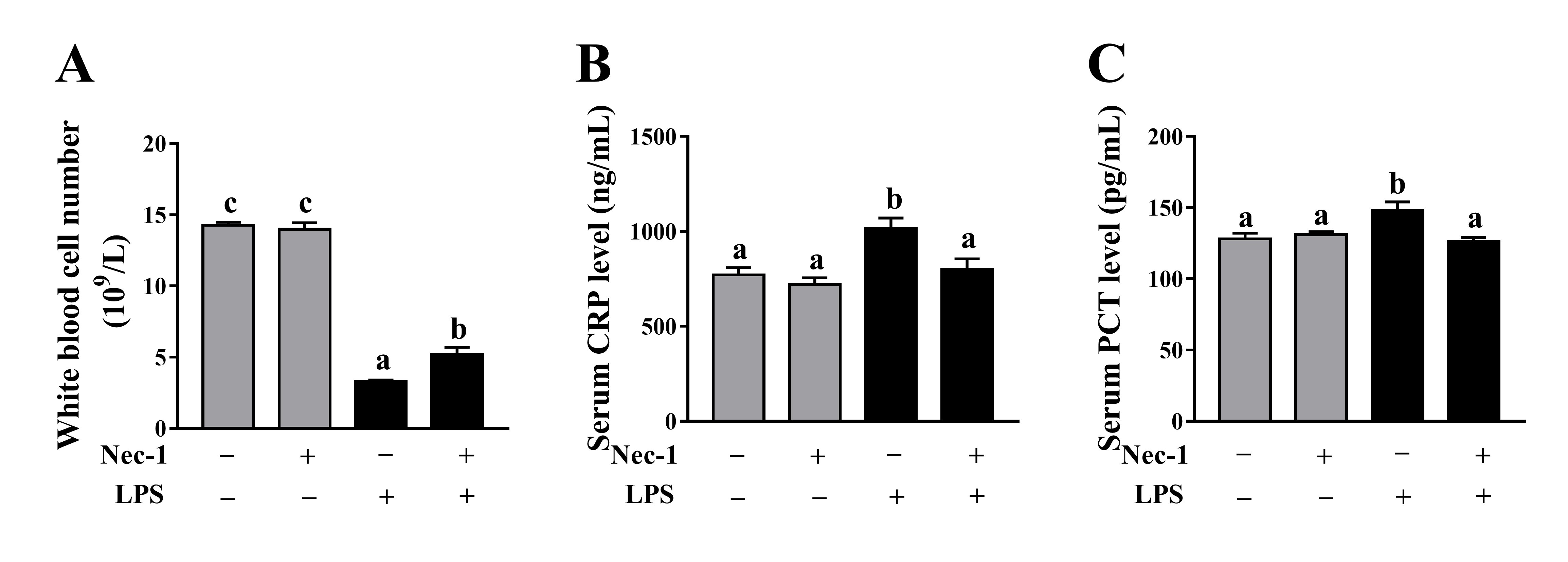

Supplement: Supplementary file 3 — Supplementary Figure 2 [file 41419_2020_3365_MOESM3_ESM.tif]
