# Supplementary material for: Necroptosis is active and contributes to intestinal injury in a piglet model with lipopolysaccharide challenge
Source: Cell Death Dis. 2021 Jan 11;12(1):62. doi: 10.1038/s41419-020-03365-1 (PMC7801412; doi:10.1038/s41419-020-03365-1)
Supplement: Supplementary file 4 — Supplementary Figure Legends [file 41419_2020_3365_MOESM4_ESM.docx]

**Fig. 1S LPS induces dynamical changes of blood biomarkers for diagnosis of sepsis in piglets.** (A) Total white blood cell count. (B) Serum C-reactive protein (CRP). (C) Serum procalcitonin (PCT). The pigs in control group were sacrificed at 0 h after injection with NaCl solution. The pigs in LPS groups were injected with LPS at 100 μg/kg body weight, and then were sacrificed at 1, 2, 4, 8, 12, or 24 h after LPS challenge. Values are means ± SE, n = 6. ****p* < 0.001, ***p* < 0.01 and **p* < 0.05, significantly different from the control group (0 h).

**Fig. 2S Inhibition of necroptosis by Nec-1 attenuates the changes of blood biomarkers for diagnosis of sepsis in piglets.** (A) Total white blood cell count. (B) Serum C-reactive protein (CRP). (C) Serum procalcitonin (PCT). The pigs were pretreated intraperitoneally with Nec-1 at 1.0 mg/kg body weight or equal volume of 2% DMSO solution 30 min before the intraperitoneal injection of LPS or saline, and the pigs were sacrificed at 4 h after LPS or saline injection. Values are means ± SE, n = 7. ^abc^Means without a common letter differ, *p <* 0.05.
